# On the Clinical Study of the Heart-Sounds

**Published:** 1858-12

**Authors:** Austin Flint


### BUFFALO MEDICAL JOURNAL

AND

### MONTHLY REVIEW.

**VOL. 14.** DECEMBER, 1858. **NO. 7.**

### ORIGINAL COMMUNICATIONS.

ART. I.—*On the Clinical Study of the Heart-Sounds.* By Professor Austin Flint, M. D.

Letter No. II.

Prof. Fenner:

Dear Sir,—In my former letter I considered, *first*, of the results of the clinical study of the *second* or *diastolic* sound of the heart in health, those which are of importance as preliminary to the study of the abnormal modifications of this sound; and, *second*, the diagnostic value of the latter as based on clinical observation in cases of cardiac disease. I propose in this letter to consider the *first* or *systolic* sound in the same points of view, i. e., physiologically, as studied by means of auscultation in health; and pathologically, as furnishing, by its abnormal modifications, physical signs of disease. This sound is less simple than its fellow, and the agencies concerned in its production are not generally regarded as so well established. It is more liable to morbid alterations, and, in short, as a subject of investigation, it is more complicated, but, at the same time, more interesting and important. Without farther introduction I will enter on its consideration.

*First or Systolic Sound of the Heart in Health and Disease.*

Auscultation in health furnishes abundant proof that this is not, like the second sound, unmixed; in other words that it consists of more than one element. Its distinctive characters, as generally described, relate to the sound heard at the point within the præcordia where its intensity is greatest, viz., over the apex of the heart. At this point, especially if the person auscultated be in the sitting posture, the first sound, as compared with the second, is more intense or accentuated; it is nearly twice as long in many persons; it is lower in pitch; the valvular quality is less apparent and may be absent; it has a booming, impulsive quality, sometimes accompanied by a slight shock, and sometimes by a faint ringing intonation, or tinnitus. These are distinctive traits determined by noting and analyzing the results of twenty-four careful examinations in a series of healthy persons. They are present and more or less marked when the stethoscope is placed over the point of the apex-beat. They are also present, but not as strongly marked, on auscultating over the body of the heart, within the superficial cardiac region. But if the stethoscope be carried to a point somewhat removed from the left border of the heart, viz., over or a little without the left nipple, (in the male) the sound is divested of some of the more striking of its distinctive characters. It is shortened, being scarcely, if at all, longer than the second sound; the interval between the first and second sound is proportionately lengthened; the intensity of the sound is notably lessened, the second sound being relatively more intense or accentuated; it is valvular in quality, resembling in this respect the second sound, but is lower in pitch, having lost the booming, impulsive quality which is apparent over the apex.* The same points of difference are observed if auscultation be practised, first, with the stethoscope applied upon the naked chest, over the apex, and, afterward, with several thicknesses of soft cloth placed between the instrument and the skin. What is the explanation of this difference? Over the apex an element enters into the first sound which is eliminated when the instrument is carried beyond the heart, or when folds of cloth are interposed. What is the element eliminated? What is the element which remains after the elimination? Auscultation in health supplies data for

---

* It should be stated that the examinations on which these statements are based, were made with Cammann's stethoscope, an instrument which, in the opinion of the writer, possesses great advantages over the ordinary wooden cylinder.

answers to these questions. Answering the second question first, the residual sound is purely valvular. It is identical in quality with the second sound. It consists, then, of an unmixed *valvular element.* The clinical study of the sound in health and disease, seems to me to furnish proof that the characters eliminated are due to the impulsion of the heart's apex against the walls of the chest. Attributing these characters mainly to this impulsion, I am led to distinguish, as the second element entering into the first sound of the heart, an *element of impulsion.* The first sound is thus composed of two elements, viz., a valvular element and an element of impulsion.

What is the proof that the characters distinctive of the first sound as heard over the apex, are due to the impulsion, against the thoracic walls, of this portion of the organ. These characters vary in their degree of prominence when auscultation is practiced on the same person in different positions, the variations corresponding to alterations of the force of the apex-beat. After studying the characters of the sound in a healthy subject in the sitting posture, let him take a recumbent position, on the back; the characters referable to impulsion are notably less marked, and, at the same time, the force of the apex-beat is diminished. Let the person next incline the body to the right side; the characters due to impulsion, are still less marked, while the apex-beat is feeble and may not be felt. Turning to the left side, the characters of the sound are more strongly marked than in the sitting posture, and the apex-beat is also more forcible. In certain abnormal conditions, the heart is prevented from coming into contact with the thoracic walls. This occurs in pericarditis with effusion, in cases of liquid effusion into the left pleura, and of emphysema affecting the left lung. Under these circumstances, the element of impulsion is lost; the first sound is short and valvular like the second. The same result is observed when the muscular power of the heart is greatly reduced, as in some cases of typhus and typhoid fevers, dilatation of the heart, fatty degeneration, etc. In these affections the first sound approximates, more or less, to the second sound as regards shortness and quality. The element of impulsion is wanting in the first sound of the fœtal heart auscultated over the abdominal walls. It may be said that this proof does not exclude the hypothesis of a sound of the muscular contraction entering into the first of the heart-sounds. Without discussing this hypothesis, which I think is disproved by auscultating the naked heart, I will only remark that, so far as the practical bearings of the analysis of the first sound are concerned, it is not a matter of much importance whether the characters attributed to impulsion be due to this cause, or more directly to muscular contraction. Regarding the former explanation as the correct one, I shall continue to designate the element, an element of impulsion.

The element of impulsion predominates, and, as it were, drowns the valvular element, over the apex and body of the heart. There are occasional exceptions to this rule in healthy persons, and in these instances the second sound may be accentuated even over the apex. At the base of the heart, frequently the valvular element predominates. At the right and inferior borders of the heart the valvular element often predominates. At any point removed from the præcordial region, to which the first sound is propagated, the element of impulsion is eliminated, leaving the valvular element. The latter, therefore, is alone diffused beyond the limits of the heart, notwithstanding it is generally drowned in the preponderating element of impulsion over the apex and body of the organ. The valvular element has less intensity and is less diffused than the second sound; for, on carrying the stethoscope away from the heart, the first sound ceases, as a rule, to be appreciable sooner than the second sound; and, as just stated, at a distance from the præcordia, the second sound becomes accentuated.

The analogy between the valvular element of the first sound and the second sound, as regards quality, leads to the inference that this element is due to the action of the auriculo-ventricular valves. Clinical observation in cases of disease, confirms the correctness of this conclusion. Hence the propriety of designating it the valvular element. Assuming the correctness of this explanation, the element consists of the action of two sets of valves, viz., the mitral and tricuspid; now, can the sound emanating from these two sources be disconnected and distinguished from each other, as the aortic and the pulmonic second sound are discriminated in certain situations? Over the inferior border of the heart, near the xiphoid cartilage, the valvular element of the first sound frequently differs in pitch from the same element when the stethoscope is applied at or without the left nipple. When this disparity in pitch is observed, it may be inferred that the predominating sound in the latter situation originates at the mitral, and in the former situation at the tricuspid valves. When this disparity exists, the pitch is lower at the inferior border of the heart. For convenience of reference, the sound near the xiphoid cartilage may be called the tricuspid valvular element, and the sound at or without the left nipple, the mitral valvular element of the first sound of the heart.

The decomposition of the first sound, and the facts which have been stated with respect to the two elements, respectively, which compose it, enable the clinical observer to understand the abnormal modifications which this sound presents in different cardiac affections. Both elements may be modified by disease, but in unequal proportions; either element may be affected independently of the other; either may be suppressed while the other remains; and of the two components of the valvular element, viz., the mitral and the tricuspid, the one may be lessened or suppressed, and the intensity of the other augmented, by disease. What are the pathological relations of these different abnormal modifications, or, in other words, what diagnostic significance belongs severally to the latter as physical signs of disease?

In cases of enlargement of the heart in which hypertrophy predominates, the intensity of the first sound is augmented more than that of the second sound; and this exaggeration is due mainly to the augmented intensity of the element of impulsion. This element is morbidly exaggerated disproportionately to the increased intensity of the valvular element. Hence, as all observers have remarked, the first sound in hypertrophy is abnormally dull and prolonged. This is intelligible, since the dullness and prolongation of the first sound are due to the element of impulsion. In proportion to the prolongation, the interval between the first and second sound is shortened; it may be almost or even quite inappreciable. In this effect of hypertrophy we have a feature which distinguishes the augmented intensity due to it, from that which occurs when the muscular action of the heart is increased by merely functional excitation. In the latter case, the first sound is abnormally exaggerated, but the exaggeration does not affect disproportionately the element of impulsion to the same extent. Hence, the sound is less dull and prolonged than in cases of hypertrophy. This fact has arrested the attention of observers. Disproportionate exaggeration of the element of impulsion in a marked degree, therefore, is a physical sign of hypertrophy.

On the other hand, whenever the muscular power of the heart is weakened, the intensity of the element of impulsion falls below that of health, and the abnormal feebleness of this element is greater than that of the valvular element. When the muscular power of the ventricles is greatly reduced, the element of impulsion may be suppressed while the valvular element is still appreciable, and in some instances both elements are extinguished and the first sound is wanting, while the second sound may still be heard. Abnormal feebleness of the element of impulsion, and its extinction occur in cases of enlargement of the heart in which dilatation greatly predominates. In proportion as this effect is produced, the first sound not only becomes enfeebled, but valvular in quality, and shortened, in these respects approximating to the second sound. In proportion as the sound is shortened, the interval between the first and second sound is lengthened. This change in the quality and duration of the first sound in cases of dilatation, was observed by Laennec, and was regarded by him, and also by Hope and others, as distinctive of dilatation. It is, however, only significant of weakened muscular power of the ventricles. It is not peculiar to cases of dilatation. It characterizes equally, as has been remarked by Stokes, the enfeebled first sound in certain cases of typhus and typhoid fever; also in cases of fatty degeneration, and, in short, whenever the muscular power of the organ is greatly reduced. When this condition exists, from whatever cause, the first sound becomes relatively more feeble than the second, and also short and valvular, resembling the latter sound in these particulars, but always lower in pitch. The second sound being much less affected by any of the various causes which enfeeble the element of impulsion of the first sound, is accentuated, even over the apex, when these causes are operative to much extent. The element of impulsion may be enfeebled and even suppressed in cases of hypertrophy, if, notwithstanding this lesion, the muscular power of the heart, from any cause, be greatly weakened. Abnormal feebleness and suppression of the element of impulsion, therefore, are physical signs of the muscular weakness of the heart due either to organic changes, dilatation, softening, fatty degeneration, etc., or to great functional debility of the organ. It is to be added that this element is eliminated, as has been seen already, when from effusion of liquid within the pericardial, or the left plural sac, from emphysema, or other causes, the heart is prevented from impinging during the systole against the walls of the chest.

The abnormal modifications affecting the element of impulsion, have been considered; it remains to consider those which affect the valvular element of the first sound. The intensity of this element is increased whenever the muscular action of the heart is unduly excited. In violent palpitation, both elements of the first sound are exaggerated in a ratio not notably unequal, while in hypertrophy, as has been seen, the intensity of the valvular element is not increased in pıoportion to that of the element of impulsion. Excited action of the ventricles, thus, tends to increase the loudness of the valvular element, more than the augmented, sluggishly excited muscular power in hypertrophy. As a physical sign, exaggeration of the valvular element has very little positive value. Diminished intensity or suppression of this element is of more importance in diagnosis.

The valvular element may be diminished or suppressed in conjunction with the element of impulsion. When both elements are thus similarly affected, great weakness of the muscular power of the heart is indicated, which may be due either to functional debility or organic change. The first and most marked effects of weakness of the heart, are apparent in the element of impulsion. If the weakness be considerable, this element is suppressed, leaving the valvular element still appreciable. If the weakness be very great, the latter element at length become inappreciable, and the first sound is extinct, while the second sound may still be heard. Extinction of the first sound occurs in some cases of typhus and typhoid fevers, and from this symptom, as the reader is doubtless aware, Dr. Stokes, and others, desire an indication for the free use of stimulants in these fevers and other diseases attended with extreme adynamia. It is also observed in some cases of fatty degeneration of the heart. If the two elements are enfeebled conjointly, and not unequally, it is not to be inferred therefrom that there exists any valvular lesion.

But the valvular element may be enfeebled or suppressed while the element of impulsion is as intense and more so than in health. Valvular lesions involving more or less damage or destruction, are indicated under these circumstances. Inasmuch as, of the auriculo-ventricular valves, the mitral are the seat of lesions in the vast majority of cases, the tricuspid valves as well known, being very rarely affected, it is the mitral valvular element which is enfeebled or suppressed in connection with the valvular lesions which produce modifications of the first sound. This element, it may be remarked, is to be studied in cases of disease, where it is best studied in health, viz., at or without the left border of the heart, in other words upon, or to the left of the nipple. The stethoscope is to be carried to a point sufficiently removed from this border of the heart, to eliminate the element of impulsion, the valvular sound which remains being that emanating from the mitral valves. Clinical observation goes to show that the valvular element in this situation is abnormally feeble, or suppressed, according to the damage which the mitral valves have received from lesions there seated. A murmur accompanies these lesions, not invariably, but in the vast majority of cases. This murmur points to the existence of mitra lesions, but it does not furnish information respecting the amount of injury which the lesions have occasioned. Cases are frequently met with in which a murmur referable to the mitral orifice is observed, without being accompanied by symptoms denoting much, if any disturbance of the circulation. The murmur may continue, denoting innocuous lesions, for many years.* When they are thus innocuous, the mitral valvular element of the first sound will be found to retain nearly or quite its normal intensity. On the other hand, when the lesions have occasioned more or less damage to the valves, the valvular element is feeble in comparison with the element of impulsion, and may be inappreciable, while the latter is not only present but perhaps exaggerated. The valvular element of the first sound, referable to the mitral valves, therefore, is to be observed in connection with mitral murmurs, as a means of determining whether, or not, and to what extent, these valves are damaged. Practically, these are the important points involved in the diagnosis of mitral lesions; for these lesions are serious, and otherwise, in proportion as the valves are injured. The presence of a murmur, it must be confessed, interferes, to a greater or less extent in some cases, with the study of the valvular element. If the murmur be intense, and especially if it be rough, the valvular element may be obscured or drowned by it. In drawing conclusions from its indistinctness or absence, when a coëxisting murmur is loud or harsh, therefore, we are not to be so positive as we may be in concluding that the integrity of the valves is not greatly compromised when the element is found to retain nearly or quite its normal intensity. It is also to be borne in mind that feebleness and suppression of the valvular element, are not physical signs of valvular injury except when the element of impulsion is not proportionately feeble or wanting. It may be added, that, when they are physical signs of valvular injury, a mitral murmur is present, as a rule to which there are a very few exceptions.

Feebleness and suppression of the tricuspid valvular element, are of no value as physical signs, for in the first place, it is only in a certain proportion of persons in health, that this portion of the valvular element can be distinguished from the portion referable to the mitral valves; and in the second place, lesions of the tricuspid valves are exceedingly infrequent. Abnormal intensity of the valvular sound emanating from the tricuspid orifice, however, is a sign of some value. This sound in disease is, of course, to be sought for in the same situation as in health, viz., at, or just below the inferior border of the heart, near the xiphoid cartilage. In some cases the valvular element in this situation is strongly marked, exceeding the intensity of the mitral sound. This occurs as an effect of hypertrophy of the right ventricle, generally resulting from mitral lesions which, at the same

* For cases illustrative of this statement, see Essay in Transactions of American Medical Association, by the writer, vol. xi, 1858.

time, lessen the intensity of the mitral valvular element. Its significance, as a physical sign, is precisely that of intensification of the pulmonic second sound of the heart. Exaggeration of the tricuspid valvular element of the first sound and of the pulmonic second sound of the heart, will be likely to be associated; but I have met with instances in which the former coëxisted with hypertrophy of the left ventricle, while the latter was not well marked. It may be remarked here, that comparison of the valvular element referable to the mitral valves, with that referable to the tricuspid valves, constitutes a means of judging, on the one hand, whether the power be abnormally weakened; or, on the other hand, whether the latter be exaggerated.

In conclusion, the practical points pertaining to the diagnostic significance of the abnormal modifications of the heart-sounds, are embodied in the following series of propositions:

1. Increased intensity of the first sound, the two elements composing this sound being affected equally, is a sign of excited, muscular action of the heart, and is observed in cases of functional disorder without organic disease.

2. Increased intensity of the element of impulsion in the first sound, the intensity of the valvular element not being proportionately augmented, if at all, is a sign of hypertrophy affecting the left ventricle.

3. Diminished intensity of the element of impulsion is a sign of weakened muscular power of the left ventricle, either from organic affections, such as dilatation, or fatty degeneration, or from functional debility of the organ. Cases are to be excluded in which, from the presence of liquid effusion in the pericardium or pleura, or from emphysema, the heart is prevented from coming into contact with the thoracic walls.

4. Abnormal intensity of the mitral valvular element of the first sound, is a sign of excited muscular action of the heart, and is accompanied by a corresponding increase of the intensity of the element of impulsion, as stated in prop. 1st. Abnormal weakness and suppression of this element, the element of impulsion retaining or exceeding its normal intensity, are signs of more or less injury of the mitral valves. A murmur referable to the mitral orifice coëxists in the vast majority of cases. Notwithstanding the murmur, if the valvular element of the first sound referable to the mitral valves, retain nearly or quite its normal intensity, the valves are not seriously damaged. In judging of the normal intensity of the mitral valvular element, it may be compared with the sound emanating from the tricuspid valves.

5. Abnormal intensity of the valvular element referable to the tricuspid valves, is a sign of hypertrophy of the right ventricle, and is generally associated with diminished intensity of the valvular element referable to the mitral valves. Abnormal weakness of the tricuspid valvular element is not available as a physical sign of disease.

6. A *positive* increase of the intensity of the pulmonic second sound of the heart, is a sign of hypertrophy of the right ventricle, in the majority of cases dependent on mitral contraction or insufficiency, or both. A *relative* increase of this sound, i. e., as compared with the aortic second sound, may result from abnormal feebleness of the aortic sound, due to mitral obstruction or regurgitation.

7. Abnormal intensity of the aortic second sound, is not a sign of much importance. But non-diminution of its intensity, in cases in which a murmur referable to the aorta is present, is a sign of much value, indicating that, although aortic lesions exist, the integrity of the valves is not seriously compromised.

8. Diminished intensity of the aortic second sound, in cases in which a murmur referable to the aorta is present, is a sign that the aortic valves are damaged, provided that neither mitral obstruction nor regurgitation exists, an effect of the latter being abnormal feebleness of this sound. If the diminished intensity of the aortic sound be due to injury of the valves of the aorta, there will be likely to be present an aortic regurgitant murmur, in other words, a diastolic murmur referable to the aorta.

9. In cases in which a diastolic murmur is present, referable either to the direct current of blood through the mitral orifice, or to aortic regurgitation, a normal intensity of the aortic second sound is evidence that the lesions giving rise to the murmur are seated at the mitral orifice.

Venturing to hope that the *résumé* which I have endeavored to present of the results of clinical study of the heart sounds in health and disease, has not been without interest for some of the readers of the Medical News and Hospital Gazette, I remain, with much respect, truly yours, A. F.

